# Size distribution of microbial aerosols in overground and subterranean treatment chambers at health resorts

**DOI:** 10.1007/s40201-020-00559-9

**Published:** 2020-10-14

**Authors:** Rafał L. Górny, Krzysztof Frączek, Dariusz R. Ropek

**Affiliations:** 1 Laboratory of Biohazards, Department of Chemical, Aerosol and Biological Hazards, Central Institute for Labour Protection–National Research Institute, Czerniakowska 16 Street, 00-701 Warsaw, Poland; 2 Department of Microbiology and Biomonitoring, University of Agriculture, Cracow, Poland

**Keywords:** Bioaerosol, Size distribution, Air quality, Sanatorium, Heath resort, Inhalation therapy

## Abstract

**Purpose:**

to perform comparative analyzes of the size distributions of bacteria and fungi in the air of overground therapy chambers in Szczawnica sanatorium and subterranean inhalation chambers in Bochnia Salt Mine health resort taking into account influence of the season and presence of pathogenic species.

**Methods:**

bioaerosol samples were collected using 6-stage Andersen impactor. Bacterial and fungal aerosol concentrations and size distributions were calculated and isolated microorganisms were taxonomically identified based on their morphological, biochemical, and molecular features. Results: in both treatment rooms and atmospheric (outdoor) air, the acceptable microbial pollution levels were periodically exceeded. The size distribution analyzes revealed that in the case of bacteria – emission from the patients and in the case of fungi – transport with atmospheric (outdoor) air were the major processes responsible for microbiological contamination of indoor premises. The majority of microbial particulates were present in the air of studied premises as single bacterial vegetative cells, spores and fungal conidia or (most commonly) formed small microbial or microbial-dust aggregates. This phenomenon may have a significant effect on patients’ actual exposure (especially on those treated for respiratory diseases) in terms of the dose of inhaled particles.

**Conclusions:**

the microbiological quality of the air in sanatoriums and health resorts is a key factor for their therapeutic and prophylactic functions. When microbial pollution crossed the acceptable level, the measures that enable reducing undesirable contamination should be introduced, especially if large groups of patients undergo such therapy.

## Introduction

Sanatoriums and health resorts are important parts of the health care system in many countries. Different treatments available there help to strengthen patient’s immune system against numerous diseases. In such places, the pharmacotherapy is often supported by the use of different health stimulants in the form of inhalations, through drinking of mineral waters or simply exposure to specific climatic conditions. Unique in this respect are therapeutic conditions characteristic for natural overground and subterranean treatment chambers [1]. Proper environmental quality is the basic criterion for therapeutic and prophylaxis functions of such places. As additional (sometimes simply occasional) exposure to pathogenic or even saprophytic microorganisms can strengthen the adverse outcomes, the microbial quality of the air is a key factor especially in the premises where patients are subjected to inhalation therapy.

Bioaerosols are defined as naturally occurring or artificially produced particulates of biological origin dispersed in the air [2–4]. These aerosols may contain culturable, nonculturable or dead microorganisms (including bacteria, fungi, and viruses), their fragments, metabolites (e.g. toxins, enzymes), and particulate waste products from all varieties of organisms [4, 5]. They can cause infections, allergic, toxic, and numerous non-specific reactions in exposed individuals [6]. Bioaerosols can also act as vectors for deep-lung and environmental transport of airborne infectious diseases [7].

The behavior of airborne microbial particles strongly depends on their physical characteristics [8, 9]. Aerodynamic diameters of microbial aerosol particles present in the environment range from nanometric values (e.g. bacterial endotoxins), through submicrometer sizes (e.g. fragments of fungal and bacterial cells), up to tens of micrometers (e.g. microbial-dust aggregates) [2, 3, 5]. Particle sizes depend among others on the air humidity and solar ultraviolet radiation, so they are subjected to changes with time of day and season [10, 11]. In practice, microbiological material is rarely present in the air as separate independent particles and quite often creates aggregates with other biological or non-biological artefacts. In many cases, microbiological particles are carried in the air by dust particles [12] or fibers [13]. Shaffer and Lighthart [14] found that the majority of particulates in terrestrial environment has aerodynamic diameters larger than 3 μm. This observation was confirmed using remote measurement data by Ho [15], who concluded that microbial particles tend to form aggregates larger than 2.5 μm in diameter. Investigations carried in indoor environment also showed that particles with smaller aerodynamic diameters were rather unable to carry intact microbial cells but were able to transport e.g. bacterial endotoxins [16].

Microbial aerosols enter the human body mainly through the nose and mouth. In the respiratory tract, the penetration depth and behavior depend on their sizes, shapes, densities, electrical charges, chemical composition, and reactivity. Moreover, the physiological factors such as airflow and breathing patterns influence the mechanism of particle deposition as well [17]. Most particles greater than 10 μm, and up to 80% of particles between 5 and 10 μm, are trapped in the nasopharyngeal region due to inertial impaction, centrifugal condensation, and interception (if resembling fibers like e.g. chains of fungal conidia) resulting from the anatomic formation of these parts of the respiratory tract where the air stream has the highest velocity. If trapped there, they may cause eye or nose irritations and asthmatic reactions, respectively. Particles smaller than 5 μm and up to 0.5 μm are deposited by sedimentation and impaction, which take place in bronchi, bronchioles, and alveoli, where the air velocity is low and the probability of deposition is directly proportional to the residence time. Particles of less than 0.5 μm are mainly separated from the air stream and deposited almost solely by diffusion. All of them, reaching lower respiratory tract, may induce allergic alveolitis types of reactions. In practice, all particles smaller than 2.5 μm (e.g. bacterial vegetative cells, actinomycete spores, most fungal conidia), can easily deliver potentially dangerous substances deep into the respiratory system, causing damage on cellular level [9, 18–20].

Understanding of particle deposition is important in assessment of the health risk of particulate air pollutants as well as in evaluation of the efficacy of therapeutic aerosols. When particle-lung interactions are studied, most frequently, an aerosol is characterized by its particle size distribution. Size distribution of microbial aerosols in health resort premises has not so far been investigated. Hence, the major objective of this study was to perform comparative analyzes of the size distributions of bacteria and fungi in the air of overground therapy chambers at the Szczawnica sanatorium and subterranean inhalation chambers at the Bochnia Salt Mine health resort. From among different environmental factors having an effect on airborne microbiota, an influence of the season and presence of pathogenic species in the air of investigated premises, as the parameters crucial from the viewpoint of health status of exposed individuals, were also considered in this study.

## Materials

The study was carried out in overground therapy chambers in Szczawnica sanatorium and in subterranean inhalation chambers in Bochnia Salt Mine health resort, both located in southern Poland. Szczawnica is an old spa town well-known for its healing microclimate with high insolation and relatively low precipitation levels. Its sanatorium specializes in medical treatment of chronic pneumonia, asthma, respiratory allergies and infections, chronic obstructive pulmonary disease, bronchiectasis, sinusitis as well as arteriosclerosis and osteoporosis. In turn, Bochnia Salt Mine health resort is a part of one of the oldest salt mines in Poland (the beginnings of the mine as an excavating plant date back to 1248). In 1995, several of its subterranean chambers were converted into a sanatorium. Till now, all of them have been using for medical treatment of respiratory tract diseases such as recurrent infections of the upper and lower airways, chronic inflammation of the pharynx and larynx, asthma, chronic bronchitis, and bronchiectasis.

The measurements of microbiological aerosols in Szczawnica sanatorium and Bochnia Salt Mine health resort were carried out over a period of one year, twice in each of four seasons. In Szczawnica, sanatorium was situated in wooded park. The air samples were collected in 3 buildings: ‘nature treatment institute’, ‘inhalatorium’, and ‘pump-room’. All the studied premises were naturally ventilated and treatment rooms were free from decorative plants. The floors in studied premises were covered with ceramic tiles or finish with polyvinyl chloride (PVC) covering. The walls were wainscoted (usually bottom half of the wall) and/or covered with washable paint. In each of these buildings, the samples were taken (in triplicate) in rooms during the treatment courses with patients and in the same rooms after curative treatment without them. In addition, in each of the investigated buildings, indoor background concentrations of bacterial and fungal aerosols were established. The rooms, where no curative treatments were present, were selected for this purpose. Moreover, in front of the ‘nature treatment institute’ building, outdoor air samples were also collected to obtain data on the background (atmospheric) level of microbial contaminants.

In Bochnia, the air samples were collected in two subterranean inhalation chambers: Ważyn and Kołdras. In each of these chambers, the samples were taken according to the schedule used in Szczawnica, i.e. during the treatment courses with patients and in the same premises after curative treatment without them. All these premises had rock-salt walls and ceilings, parquet or tile floors and were free from decorative plants. Moreover, the air samples were also taken from the ramp, an incline of 139 m long being a part of the rock-salt corridor connecting Ważyn and Kołdras chambers. Here also, for each of the investigated areas, indoor background concentrations of bacterial and fungal aerosols were established. All these measurements were carried out in the galleries located on the same levels as the studied chambers, through which the air was introduced into them. The mechanical ventilation system placed above the ground in a slightly afforested area was responsible for a delivery of the atmospheric air into the studied subterranean chambers. In order to obtain data on the background atmospheric level of microbial contaminants, the air was sampled in front of an overground fan.

## Methods

All bioaerosol samples were collected using a 6-stage Andersen impactor with a pump (model 10–710, Graseby-Andersen, Inc., Atlanta, GA, USA) enabling a division of bioaerosol particles into six fractions according to their aerodynamic diameters, d_ae_, i.e. above 7, 7–4.7, 4.7–3.3, 3.3–2.1, 2.1–1.1 and 1.1–0.65 μm. The Andersen sampler was always placed at a height of 1–1.5 m above the floor or ground (outdoor measurement) level to simulate aspiration from the human breathing zone. Each time, a 5-min sampling period was applied for the collection of bacterial and fungal aerosols. Samples were taken at a flow rate of 28.3 L min^−1^. Bacteria were collected first, on blood trypticase soy agar (TSA; Becton, Dickinson and Company, Sparks, MD, USA). After impactor reloading, the fungi were collected on malt extract agar (MEA; Oxoid Ltd., Basingstoke, Hampshire, UK). During sampling, the air temperature and relative humidity (RH) were controlled using a hytherograph (model Omniport 20, E + E Elektronik GmbH, Engerwitzdorf, Austria). After sampling, the TSA plates were incubated for 1 day at 37 °C followed by 3 days at 22 °C and another 3 days at 4 °C, and MEA plates for 4 days at 30 °C followed by 4 days at 22 °C. After incubation of the plates, the quantitative and qualitative analyzes of culturable microorganisms were performed. The concentration of bioaerosols was calculated as colony forming units per cubic meter of the air (CFU m^−3^). Bacterial strains were identified by Gram staining, their morphology and, finally, by the biochemical API tests (bioMérieux, Marcy l’Etoile, France). Fungi were identified according to their morphology using several identification keys [21–24].

The most prevalent microbial isolates and pathogens were additionally analyzed by molecular methods (polymerase chain reaction (PCR) followed by random amplification of polymorphic DNA-RAPD typing). DNA was isolated from pure bacterial/fungal cultures grown on TSS/MEA plates using QIAmp DNA (Qiagen, Hilden, Germany) or Fungi DNA (Syngen Biotech, Wrocław, Poland) Mini Kits. The isolated bacterial DNA was used as a template in PCR with BAK11w (5′-AGTTTGATCMTGGCTCAG-3′) and BAK2 (5′-GGACTACHAGGGTATCTAAT-3′) primer sets which allow amplification of bacterial 16S rRNA gene fragment corresponding to *Escherichia coli* 16S rRNA gene positions from 10 to 806. The isolated fungal DNA was used as a template in PCR with ITS1 (5′-TCCGTAGGTGAACCTGCGG-3′) and ITS4 (5′-TCCTCCGCTTATTGATATGC-3′) primer sets which allow amplification of fungal genome fragment located between 18S and 28S rRNA genes, covering ITS1, 5.8S rRNA, and ITS2 fragments. The amplified PCR products were purified, sequenced using DNA analyzer (model 3730, Applied Biosystems, Waltham, MA, USA), and compared to GenBank database (National Center for Biotechnology Information, U.S. National Library of Medicine, USA) using BLAST (Basic Local Alignment Search Tool) algorithm [25–27]. In total, 3 bacterial and 5 fungal species were analyzed in that way.

All collected data were statistically elaborated using analysis of variance (ANOVA) followed by Scheffé test, *t*-test and Pearson correlation analysis using Statistica (data analysis software system) version 10.–2011 (StatSoft, Inc., Tulsa, OK, USA).

## Results and discussion

**Fig. 1:**
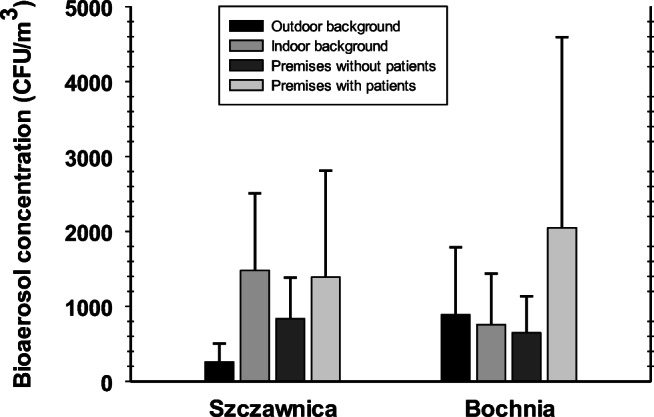
Annual mean concentrations (with standard deviations) of total bioaerosol (bacteria and fungi together) measured at Szczawnica sanatorium and Bochnia Salt Mine health resort

**Fig. 2:**
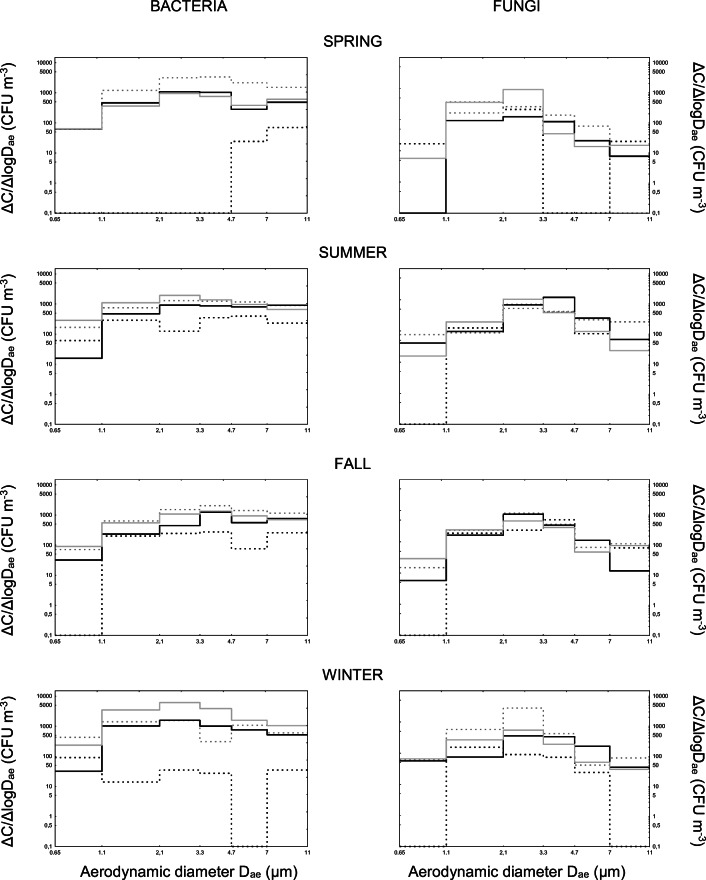
Size distribution of microbial aerosols at Szczawnica sanatorium. The line patterns represent microbial concentrations in the air of: outdoor (dashed black) and indoor (dashed grey) backgrounds as well as premises without (solid black) and with (solid grey) patients

**Fig. 3:**
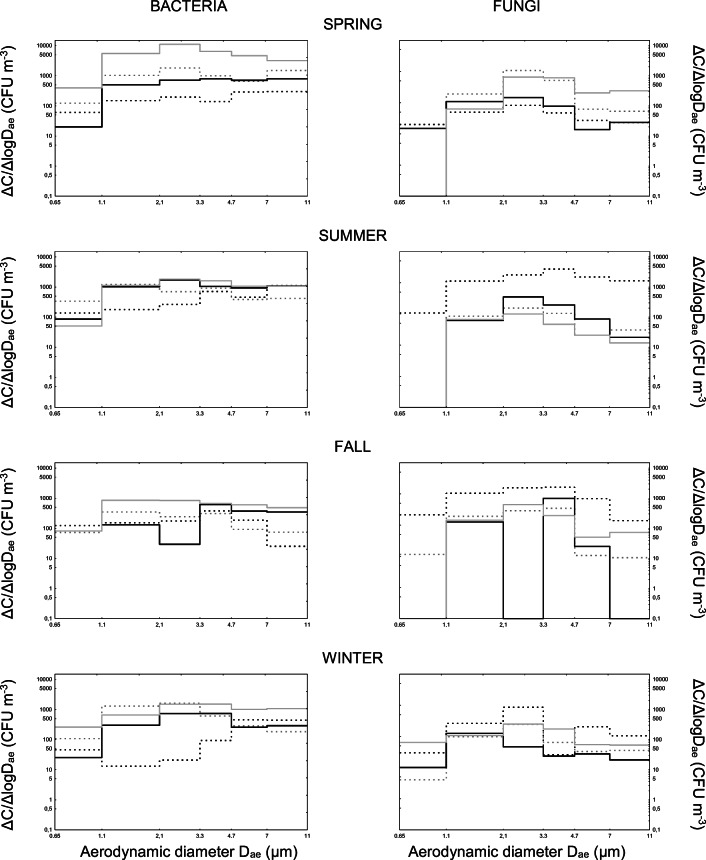
Size distribution of microbial aerosols at Bochnia Salt Mine Health Resort. The line patterns represent microbial concentrations in the air of: outdoor (dashed black) and indoor (dashed grey) backgrounds as well as premises without (solid black) and with (solid grey) patients

The results of quantitative and qualitative analyses of airborne microbiota sampled in Szczawnica sanatorium and Bochnia Salt Mine health resort are presented in Tables 1, 3 and 4 as well as in Figs. 1, 2 and 3. The maximal indoor concentrations of bacterial and fungal aerosols in Szczawnica as well as Bochnia reached 6223 CFU m^−3^ and 1575 CFU m^−3^ as well as 11,668 CFU m^−3^ and 566 CFU m^−3^, respectively. The respective outdoor levels amounted to 366 CFU m^−3^ and 352 CFU m^−3^ as well as 2517 CFU m^−3^ and 9731 CFU m^−3^, respectively. Considering all seasons together, a comparison of bacterial and fungal concentrations in outdoor background revealed generally significantly higher contamination levels of atmospheric air in Bochnia than in Szczawnica (*t*-test: *p* < 0.05 and *p* < 0.01, respectively). In turn, in case of bioaerosol concentrations recorded in indoor background, fungal levels followed the outdoor trend, whereas an opposite relationship was observed for airborne bacteria, i.e. their concentrations in Szczawnica were significantly higher than those in the indoor background in Bochnia (*t*-test: p < 0.01). The analysis of microbial aerosol concentrations in treatment rooms against background bioaerosol levels showed that in Szczawnica sanatorium, in case of bacteria, the patients during treatment procedures are an important source of bacterial emissions into the air. This is particularly noticeable in summer and winter seasons when the natural ventilation of treatment premises decreases in a natural way (in the summer to keep the indoor temperature at lower level than outdoor one and in the winter to isolate the naturally aired room from the cold outdoor air) (Scheffé test: in both cases *p* < 0.05). After performing therapeutic activities, the treatment rooms are effectively cleaned and the level of microbiological impurities decreases to the level of the internal background. Indoor microbial contamination, regardless of the season and purpose of a given room, was always significantly higher than the pollution of external (atmospheric) air (Scheffé test: p < 0.05). In turn, in the case of fungal aerosol, the presence of patients does not significantly increase the number of fungi in the air. However, in relation to these microorganisms, the hygienic condition of the rooms after the performed therapeutic procedures may be a problem. The treatment rooms are well insulated and especially in warmer seasons (in this case in summer and fall), these rooms should be better ventilated or otherwise cleaned in order to remove the fungal aerosol particles accumulated in them after treatment of the patients.

**Table 1:** Bacterial and fungal aerosol concentrations (CFU m^−3^) at Szczawnica sanatorium and Bochnia Salt Mine health resort

Environment	Season	Szczawnica premises				Bochnia premises			
		Bacteria		Fungi		Bacteria		Fungi	
		Average	Range	Average	Range	Average	Range	Average	Range
Rooms during treatment courses with patients	Spring	521	137–947	227	18–933	5207	595–11,668	301	62–566
	Summer	1054	287–1943	292	46–1247	1123	322–1814	40	7–99
	Fall	779	290–1335	193	29–473	587	381–849	149	41–454
	Winter	2711	234**–**6223	192	30–545	1001	560–1509	116	52–173
Rooms after treatment courses without patients	Spring	567	62–1078	56	0–125	588	35–796	64	13–108
	Summer	667	21–918	389	40–1109	985	86–2409	116	17–213
	Fall	543	31–1233	235	5–771	262	49–612	144	20–226
	Winter	815	32–1560	171	35–358	389	45–737	41	7–125
Indoor background	Spring	1918	671–3369	105	35–164	1019	122–1815	316	51–367
	Summer	920	687–1290	255	76–527	661	382–1218	65	60–154
	Fall	1140	771–2053	268	45–842	189	143–347	143	69–346
	Winter	896	706–1552	661	41–1575	680	207–1302	80	54–246
Outdoor background	Spring	24	16–70	103	14–268	189	61–300	41	28–82
	Summer	242	61–366	252	80–352	473	189–2517	1411	107–9731
	Fall	176	76–273	174	47–313	172	25–377	889	140–1637
	Winter	34	14–91	56	24–154	178	13–456	249	30–868

In Bochnia Salt Mine health resort, similarly like in Szczawnica, the patients served also as the main emitter of bacteria into the air of treatment rooms. After the end of therapeutic procedures, the number of bacteria in the air decreased significantly, usually to the level of the indoor background (Scheffé test: *p* < 0.05). Insulation of internal spaces, however, makes the efforts to remove bacterial aerosol by simple ventilation less effective. Regardless of the season, the level of indoor bacterial contaminants was always significantly higher than this observed in atmospheric (outdoor background) air (Scheffé test: p < 0.05). In the case of fungal aerosol, regardless of the season, atmospheric air in relation to the indoor air has always been significantly more polluted (Scheffé test: p < 0.05). The presence of patients in treatment rooms usually caused fungal contamination of these interiors to the level of indoor background. After the therapeutic procedures, the rooms were cleaned and the level of fungal air contamination declined significantly reaching the concentrations characteristic for indoor background (except for the summer season – in this case, the lack of significant decrease of airborne fungi levels was probably a result of increased mechanical ventilation during the warmest season of the year). Nevertheless, the observed trends confirm both a good isolation of the treatment areas from major contamination sources and their effective hygienic maintenance in this health resort.

On the global scale, there is a lack of acceptable exposure limits (or threshold limit values, TLVs) for indoor and outdoor bioaerosols. The main reason for this is still perceptible deficiency of well-documented and epidemiologically proven dose-response relationships between the exposure to specific biological agents and adverse health effects caused by their exact doses. Moreover, the sensitivity to each organism is its individual feature and the strength of immunological reaction to the specific agent(s) is usually not the same in everybody. Despite progress in development of aerosol sampling techniques and analytical methods over the last three decades, the worldwide scientific database on bioaerosols is still insufficient to quantitatively and qualitatively characterize them. If threshold limit or reference values are established, they are usually connected with the clinical picture of specific disease caused by the agent, taking into consideration its presence in a certain element of the environment only [28, 29]. Nevertheless, despite these limitations, the reference values expressed in numbers are available and usually applied to facilitate interpretation of the measurement data. For the purpose of this study, the observed microbial concentrations were interpreted against the available TLV proposals [30, 31]. Taking into account the TLVs established for mesophilic bacteria and fungi (i.e. 1 × 10^5^ CFU m^−3^ and 5 × 10^4^ CFU m^−3^, respectively), it can be stated that in both treatment rooms and atmospheric (outdoor) air, the acceptable pollution levels were exceeded. In the case of bacteria, these exceedances were observed in the air of treatment rooms both in Szczawnica sanatorium and in Bochnia Salt Mine health resort. In the case of fungi, the concentration values higher than permissible were measured only in the summer season in the outdoor air in Bochnia Salt Mine health resort. For obvious reasons, it is difficult to influence the environmental level of fungal contamination, which is dependent on the current prevailing atmospheric conditions. Nevertheless, the exceedances of permissible bacterial concentrations in treatment rooms are evidently the result of the emission of this type of bioaerosol particles by patients and related to the specific treatments in the therapy of respiratory diseases (e.g. inhalations).

Simultaneously with bioaerosol measurements, the environmental parameters, i.e., air temperature and relative humidity were also controlled. The results of these measurements are presented in Table 2. In Szczawnica sanatorium, only relative humidity of the air had a noticeable influence on the observed microbial concentrations. Correlation analysis revealed that each increase in moisture content in the air resulted in a significant rise of both the airborne bacterial and fungal levels (r^2^ = 0.18 at *p* < 0.05 and r^2^ = 0.21 at p < 0.05, respectively). The measurements of air temperature and relative humidity in Bochnia Salt Mine health resort revealed high stability of thermal conditions. Despite the natural fluctuations of outdoor air temperature over a year, there were no differences between the average temperature values measured indoors and those noted in outdoor background. Compared to air temperature, the changes in relative humidity within a year in the health resort chambers were more distinct and statistically significant differences in the examined premises were observed between summer and winter values (*p* < 0.01). The analyses of the influence of microclimatic parameters on the observed bacterial and fungal aerosol concentrations in Bochnia showed that only bacterial aerosol levels were affected by these factors. The correlation analysis revealed that each rise in temperature and relative humidity in the studied chambers resulted in significant increase in bacterial concentration in the air (r^2^ = 0.35 at *p* < 0.05 and r^2^ = 0.19 at p < 0.05, respectively).

**Table 2:** Temperature (°C) and relative humidity (%) of the air at Szczawnica sanatorium and Bochnia Salt Mine health resort

Environment		Temperature		Relative humidity	
		Range	Median	Range	Median
Szczawnica sanatorium	Rooms during treatment courses with patients	18–24	21	33–84	47
	Rooms after curative treatment without patients	15–26	21	32–54	44
	Indoor background	19–26	22	34–68	38
	Outdoor background	-3–27	15	36–77	54
Bochnia Salt Mine health resort	Rooms during treatment courses with patients	15–19	17	39–73	61
	Rooms after curative treatment without patients	13–19	16	25–71	55
	Indoor background	12–19	16	38–70	64
	Outdoor background	1–29	17	40–80	72

All bacterial and fungal species isolated from the air in Szczawnica sanatorium and Bochnia Salt Mine health resort are listed in Tables 3 and 4. In total, in Szczawnica sanatorium premises 19 bacterial species belonging to 10 genera and 22 fungal species belonging to 14 genera were identified. Outdoor microbiota was less taxonomically diverse, i.e. only 11 bacterial species belonging to 7 genera and 11 fungal species belonging to 8 genera were identified. The air microbiota in Bochnia Salt Mine health resort was a bit more taxonomically abundant, i.e. 25 bacterial species belonging to 12 genera and 26 fungal species belonging to 12 genera were identified indoors, whereas 15 bacterial species belonging to 9 genera and 16 fungal species belonging to 11 genera were isolated from the outdoor air samples. All the most prevalent and pathogenic microbial isolates identified using biochemical and microscopic techniques were additionally taxonomically characterized using molecular methods. In all cases, compared to GenBank database, the sequence similarity of 99–100% was achieved. Among isolated microorganisms, the most prevalent indoors were Gram-positive cocci from *Micrococcus* and *Staphylococcus* genera (often accounted for at least 50% of the microbiota isolated from a given sample) and filamentous fungi from *Cladosporium*, *Penicillium*, and *Scopulariopsis* genera. The most abundant outdoor microorganisms belong to *Micrococcus*, *Staphylococcus*, *Bacillus*, *Cladosporium*, and *Geotrichum* genera. Among isolated microbiota, bacterial pathogens from *Corynebacterium* (*C*. *xerosis*) and *Streptomyces* (*S. albus*) genera were identified. All these strains are classified in group 2 according to their level of risk of infection [32–34] and may be responsible for adverse health outcomes in exposed individuals. Taking into account their aerodynamic sizes (see below) and immunological reactivity of their cells (responsible among others for inflammations and allergic reactions in respiratory tract), their presence is undesirable in such therapeutic premises. Comparison of qualitative composition of airborne microbiota identified in Szczawnica sanatorium and Bochnia Salt Mine health resort premises with similar objects of this type in the world is difficult due to the relatively scarce data in this regard in the scientific literature. Against the background of available results in this respect, obtained e.g. in Ciechocinek and Inowrocław spas [35–37], siderite mine used for speleotherapy in Brescia [38], salt mines health resorts in Wieliczka and Polkowice-Sieroszowice [39, 40], the data collected in this study showed a much greater qualitative spectrum of airborne microorganisms. The detection of more qualitatively complex microbiota was probably possible due to the use of a high-performance analytical approach combining the air sampling using 6-stage Andersen impactor with laboratory analysis of the resulted samples using biochemical and molecular identification methods of isolated microbial strains.

**Table 3:** Microbial taxa isolated from the air at Szczawnica sanatorium

Microorganisms	Background		Premises	
	Outdoor air	Indoor air	With patients	Without patients
Bacteria				
**Gram-positive cocci**				
*Kocuria rosea*	×	×	×	×
*Micrococcus* spp.	×	×	×	×
*Staphylococcus haemolyticus*		×	×	×
*Staphylococcus hominis*	×	×	×	×
*Staphylococcus lentus*	×	×	×	×
*Staphylococcus simulans* *	×	×	×	×
*Staphylococcus* spp.			×	
*Staphylococcus xylosus*	×	×	×	×
**Non-sporing Gram-positive rods**				
*Arthrobacter* spp.		×	×	×
*Corynebacterium xerosis* *	×		×	×
*Microbacterium* spp.			×	×
**Endospore forming Gram-positive rods**				
*Bacillus cereus*		×	×	×
*Bacillus licheniformis*		×	×	
*Bacillus megaterium*	×	×	×	
*Bacillus pumilus*	×			×
*Bacillus* spp.			×	×
**Mesophilic actinomycetes**				
*Nocardia* spp.		×	×	×
*Rhodococcus* spp.	×	×	×	×
*Streptomyces albus* *	×	×	×	×
Fungi				
**Filamentous fungi**				
*Acremonium strictum*	×			
*Acremonium* spp.	×	×	×	
*Alternaria alternata*	×	×	×	×
*Alternaria* spp.		×		
*Aspergillus* spp.		×	×	×
*Cladosporium cladosporioides* *	×	×	×	×
*Cladosporium herbarum*	×			×
*Cladosporium* spp.	×	×	×	×
*Fusarium sporotrichioides*	×		×	×
*Fusarium* spp.		×	×	×
*Helminthosporium* spp.	×	×	×	×
*Penicillium chrysogenum* *			×	
*Penicillium griseofulvum*				×
*Penicillium* spp.	×	×	×	×
*Rhizopus stolonifer*			×	
*Rhizopus* spp.		×		
*Scopulariopsis* spp. *		×	×	×
*Sporotrichum* spp.	×	×		×
*Trichoderma* spp.		×	×	×
**Yeasts**				
*Candida famata*		×	×	×
*Candida* spp.	×			×
*Cryptococcus* spp.				×
*Rhodotorula mucilaginosa*		×	×	

Note: * – strain additionally identified using molecular method

**Table 4:** Microbial taxa isolated from the air at the Bochnia Salt Mine Health Resort

Microorganisms	Background		Premises	
	Outdoor air	Indoor air	With patients	Without patients
Bacteria				
**Gram-positive cocci**				
*Kocuria rosea*	×		×	
*Micrococcus* spp.	×	×	×	×
*Micrococcus luteus*		×		
*Staphylococcus capitis*	×	×	×	×
*Staphylococcus cohnii* ssp. *cohnii*	×	×	×	×
*Staphylococcus epidermidis*		×	×	
*Staphylococcus haemolyticus*	×	×		×
*Staphylococcus hominis*		×	×	
*Staphylococcus lentus*	×	×	×	×
*Staphylococcus sciuri*	×	×	×	
*Staphylococcus xylosus*	×	×		×
*Staphylococcus* spp.				×
**Non-sporing Gram-positive rods**				
*Brevibacterium* spp.	×	×	×	×
*Corynebacterium xerosis* *	×	×	×	×
*Microbacterium* spp.		×	×	
**Endospore forming Gram-positive rods**				
*Bacillus cereus*	×		×	
*Bacillus licheniformis*	×		×	
*Bacillus polymyxa*			×	
*Bacillus pumilus*			×	×
*Bacillus* spp.			×	
**Gram-negative rods**				
*Aeromonas hydrophila*		×		×
*Chryseobacterium indologenes*		×		×
**Mesophilic actinomycetes**				
*Nocardia* spp.	×	×		×
*Rhodococcus* spp.	×	×	×	×
*Streptomyces albus* *	×	×	×	×
Fungi				
**Filamentous fungi**				
*Acremonium strictum*	×			×
*Acremonium* spp.		×		
*Alternaria alternata*		×	×	
*Alternaria chartarum*				×
*Alternaria* spp.	×		×	×
*Aspergillus sydowii*				×
*Aspergillus terreus*			×	
*Aspergillus* spp.	×			×
*Cladosporium cladosporioides* *		×	×	×
*Cladosporium herbarum*	×			
*Cladosporium macrocarpum* *	×			×
*Cladosporium* spp.	×	×	×	
*Fusarium* spp.	×			×
*Fusarium solani*	×	×		×
*Mucor plumbeus*	×			×
*Penicillium chrysogenum* *			×	
*Penicillium crustosum*			×	
*Penicillium funiculosum*		×		×
*Penicillium griseofulvum*	×	×	×	
*Penicillium* spp.	×	×	×	×
*Rhizopus stolonifer*	×			×
*Rhizopus* spp.	×			×
*Sporotrichum* spp.	×	×	×	
*Trichoderma viride*			×	
**Yeasts**				
*Geotrichum candidum* *		×	×	×
*Geotrichum* spp.	×		×	
*Candida* spp.	×		×	×

Note: * – strain additionally identified using molecular method

Bioaerosol measurements performed using 6-stage Andersen impactor allowed obtaining data on the size distribution of microorganisms present in both indoor and outdoor air in all four seasons in Szczawnica sanatorium and in Bochnia Salt Mine health resorts. The obtained results are presented in Figs. 2 and 3. The analyzes of size distribution of airborne bacteria sampled in Szczawnica sanatorium revealed that, independently of the season, the outdoor bacterial concentrations within aerodynamic diameters above 1.1 μm (i.e. within the range were the presence of intact bacterial cells, spores or their aggregates with dust particles can be expected) were always significantly lower than indoor concentrations (Scheffé test: in all cases *p* < 0.05). In the spring, bacteria in outdoor air were solely present as large bacterial or bacterial-dust aggregates. Their concentrations in treatment rooms were also always lower that those noted in indoor background (regardless of whether the patients were medically treated there or were not present in studied premises). Such picture confirms a good isolation of treatment rooms from the conditions prevailing outside them. Airborne bacteria in treatment rooms were mainly present as small bacterial and/or bacterial dust aggregates between 2.1–4.7 μm in aerodynamic diameters. In the summer, bacteria were present in the air in all possible states, i.e. as single vegetative cells or spores as well as their aggregates and/or bacterial-dust aggregates. In treatment rooms, the patients were able to load the air of these premises with additional amount of small bacterial and/or bacterial-dust aggregates of 2.1–3.3 μm in aerodynamic diameters. In the fall, the situation of air contamination resembled those from the spring, i.e. the outdoor bacterial concentrations were lower than indoor ones and the concentrations in treatment rooms were lower than in indoor background. Bacteria, if present indoor, were able to form small microbial or microbial-dust aggregates of 3.3–4.7 μm in aerodynamic diameters. The winter picture in turn resembled this from the summer. In this season, a significant increase of small (2.1–3.3 μm) bacterial or bacterial-dust aggregates was visible as probably the results of both the augmented emission from patients and increase of treatment room airtight sealing forced by cold winter conditions (less frequent airing of the rooms to prevent the loss of accumulated heat).

In case of fungi, the analyses of size distribution of these airborne microorganisms collected in Szczawnica sanatorium showed that in the spring they were present in outdoor air mainly as single conidia (2.1–3.3 μm) or large fungal of fungal-dust aggregates (>7 μm). During the migration process from outdoor environment into the indoor spaces, these large structures were broken down forming smaller particulates of 3.3–7 μm, whereas single conidia were still able to freely migrate indoors. The appearance of patients resulted in additional emission of single fungal conidia or in their resuspension from surfaces with peak concentrations between 2.1–3.3 μm in aerodynamic diameters. In the summer and fall, the presence of patients in treatment rooms still resulted in formation of fungal aerosols as single conidia between 2.1–3.3 μm in aerodynamic sizes. After the treatment courses, airborne conidia were still in the air in unchanged aerodynamic form (fall) or began to combine probably with dust particles forming slightly larger (3.3–4.7 μm) aggregates (summer). In the winter, when the level of airborne conidia in outdoor air significantly decreases in natural way (due to low temperatures and snow cover), in the treatment rooms, an unequivocal domination of single conidia (2.1–3.3 μm) in the air was again visible. Their level in the rooms without patients was slightly lower and significantly smaller number of conidia was also observed compared to indoor background. Such situation clearly confirmed that the hygienic state of the treatment rooms was properly controlled and maintained.

In turn, the analyses of size distribution of airborne bacteria sampled in Bochnia Salt Mine health resort revealed that in the spring, their airborne levels were significantly lower that their indoor concentrations. Here also, the patients were the major emission source of the most prevalent small bacterial aggregates (2.1–3.3 μm) as well as single vegetative cells or spores and larger bacterial and/or bacterial-dust aggregates. After leaving the treatment rooms, the level of bacterial contamination of these premises was falling below the indoor background level. In the summer, the patients were still the main emission source of single bacterial cells or spores and small bacterial and/or bacterial-dust aggregates into the indoor air (the peak concentrations between 2.1–4.7 μm in aerodynamic diameters). However, compared to other seasons, their concentrations, even when the patients already left the treatment rooms, still remained at a similar level (especially regarding single vegetative cells, spores or small bacterial aggregates with d_ae_ of 1.1–3.3 μm). In the fall, the patients remained the major emitters for bacterial particulates in the form of single cells or spores and different size aggregates of bacterial and/or bacterial-dust origin. While the patients’ exit from the treatment rooms did not affect the number of bacterial aggregates in the air, the absence of patients significantly lowered the number of single bacterial cells or spores and small bacterial aggregates (1.1–3.3 μm) in those premises. Similar phenomenon was observed in the winter for particles with d_ae_ characteristic for single vegetative cells or spores (2.1–3.3 μm), small (2.1–4.7 μm) and large (>7 μm) aggregates.

The analyses of size distribution of airborne fungi collected in Bochnia Salt Mine health resort revealed that the spring was the only season, in which their concentrations in the atmospheric (outdoor) air were lower than indoor levels. In the air of studied treatment premises, fungi were present mainly as single conidia (2.1–3.3 μm) and, along with the exit of patients from the treatment rooms, their concentrations approached to the indoor background level. In the summer, the level of fungal contamination of the treatment rooms was significantly lower than in the background (Scheffé test: *p* < 0.05). Such relationship could be explained by particularly low level of air humidity in this season. In the fall, the indoor contamination levels, although very variable, were always lower than those for outdoor air. The presence of patients (compared to rooms without them) raised the contamination level, especially regarding the single conidia (2.1–3.3 μm) and large fungal and/or fungal-dust aggregate (>7 μm) concentrations. The winter situation resembled that from the fall; however, the amplitude of concentration fluctuations in the rooms during treatment procedures and in these rooms without patients was significantly lower than in the fall (Scheffé test: p < 0.05).

The size of airborne microorganisms is a key determinant of disease transmission [41, 42]. Studies of cough aerosols and of exhaled breath from patients with various respiratory infections have shown striking similarities in aerosol size distribution, with a predominance of pathogenic strains in small aerosol fractions below 5 μm. These small particles are believed to cause respiratory tract adverse outcomes and increase severity, morbidity, and in the worst cases fatality of lung infections [41, 43]. Taking into account aerodynamic diameters of airborne bacteria and fungi from predominant microbial groups (i.e. for: *Micrococcus* d_ae_ = ~1 μm, *Staphylococcus* d_ae_ = ~0.75 μm, *Bacillus* d_ae_ = ~0.9 μm, *Corynebacterium* d_ae_ = ~0.7 μm, *Streptomyces* d_ae_ = ~0.85 μm, *Aspergillus* d_ae_ = ~2.4 μm, *Cladosporium* d_ae_ = ~1.8 μm, *Penicillium* d_ae_ = ~2.8 μm, *Scopulariopsis* d_ae_ = ~5.3 μm), the size distribution analysis allowed defining not only the forms of occurrence of studied bioaerosol particulates but possible depth of their penetration within the human respiratory tract and, by that, the place of their potential deposition. Such information is of a great importance especially for the patients who undergo medical procedures in treatment premises. Based on the above described results, the highest ‘load’ of bioaerosol particulates in indoor premises in both Szczawnica sanatorium and Bochnia Salt Mine health resort may reach: in case of bacteria – the trachea, primary and secondary bronchi, in case of fungi – the nasal and oral cavities as well as secondary bronchi. Such stimulation may be responsible for the adverse health effects in exposed individuals having a character of asthmatic and allergic alveolitis type outcomes (after bacterial aerosol stimulation) as well as nose and eyes’ irritations and allergic alveolitis type reactions (after fungal aerosol exposure) [18, 44–46].

The data on size distribution of airborne microorganisms in healthcare built environments are still scarce in the scientific literature. Noble and Clayton [47] investigating the airborne mycobiota in hospital wards using size-grading slit sampler revealed that majority of filamentous fungi (i.e. up to: 69% of *Aspergillus*, 66% of *Penicillium*, 55% of *Paecilomyces*, 42% of *Cladosporium*) conidia and even yeast (i.e. up to 57% of *Rhodotorula*) cells were present in the respirable (below 4 μm) size fraction. Coggins et al. [48] studying the airborne microbiota in podiatry clinic showed that the highest concentrations of bacteria and fungi occurred for particles with d_ae_ between 1.1–3.3 μm). A significant percentage of bioaerosol particles (reaching e.g. 87% for fungi) was observed in the respirable fraction. The authors, however, did not notice significant difference between bioaerosol concentrations measured in the morning prior to the first and in the evening after the last patient treatments. An opposite picture was observed by Tsay et al. [40] studying size distribution of bacterial aerosol in intensive care units in Taiwan hospital. The concentrations of microbial particles in the size range of 1.1–4.7 μm (including opportunistic pathogens from *Micrococcus*, *Staphylococcus*, and *Acinetobacter* genera) during the patient visits were three to four times higher than those before their presence in intensive care units. Nasir et al. [49] studying the size distribution of airborne bacteria and fungi in two types (conventionally ventilated and with laminar flow) of orthopedic theatres found higher concentrations in the conventionally ventilated theatre than in the laminar flow one. In terms of size distribution, the most prevalent bacteria and fungi in the wards were in the size range of 2.1–3.3 μm. The bacterial concentrations in the conventionally ventilated operating theatre were mostly in coarse size fraction compared to fine fraction for the theatre with laminar flow. Kim et al. [50] studying microbial size distribution in the air of four measurement sites (main lobby, intensive care unit, surgical ward, and biomedical laboratory) in the general hospital in Seoul (Korea) revealed that the predominant bacterial species from *Staphyloccocus*, *Micrococcus*, and *Corynebacterium* genera were present in the respirable bioaerosol fraction, i.e. between 1.1–2.1 μm, whereas bacterial species from *Bacillus* genus and predominant fungal species from *Cladosporium*, *Penicillium*, *Aspergillus*, and *Alternaria* genera were noted in coarse particulate fractions >7 μm in aerodynamic diameters. In turn, Pastuszka et al. [51] checked the presence of airborne bacteria in the air of three different hospitals and one medical clinic in Upper Silesia, Poland. The measurements were performed in both naturally and mechanically (HVAC) ventilated rooms showing the peak concentrations in the aerodynamic size ranges between 1.1–3.3 μm and 3.3–4.7 μm, respectively. The representatives of *Staphylococcus*, *Micrococcus*, and *Corynebacterium* genera were a dominating part of the bacteria in studied hospitals/clinic air, contributing together up to 78% of the total bacterial biota concentration and confirming that detected airborne species mainly originated from human organisms. Such observation is in a good agreement with our knowledge about the major bioaerosol sources in the built environment as humans carry ~10^12^ and ~ 10^14^ microorganisms on their epidermis and in alimentary tract, respectively. Both respiration and shedding of skin cells significantly contribute to the total number and community structure of bioaerosols, especially in poorly ventilated or heavily occupied environments [52–55].

Against this background, the results of the measurements performed in Szczawnica sanatorium and in Bochnia Salt Mine health resort follow the above described relationships regarding the size distributions of the most prevalent microbial representatives in the air of studied premises. Aerodynamic diameters of bacterial particles from studied subterranean chambers and sanatorium buildings confirm their significant emission from the patients. In the case of fungi, their transport with atmospheric (outdoor) air is the major process responsible for mycological contamination of indoor premises. The majority of microbial particulates are present in the air of studied premises as single bacterial vegetative cells, spores and fungal conidia or (most commonly) form small microbial and/or microbial-dust aggregates. This phenomenon may have a significant effect on patients’ actual exposure (especially on those treated for respiratory diseases) in terms of the dose of inhaled particles [3, 5, 6, 17, 56]. According to Brągoszewska et al. [57], the number of viable airborne microorganisms may reach up to 85% of the total respirable particles and, by that, significantly increase the risk of appearance of adverse health outcomes among exposed individuals.

## Limitations of the study

The major limitation of this study seems to be the number of evaluated samples; however, the evaluated premises were selected as reasonably representative for studied environments. It should be also clearly stated that bioaerosol sampling using Andersen cascade impactor enables the evaluation of viable and culturable propagules including fungal conidia, bacterial spores, and vegetative cells. Such an approach omits the health significance of viable and non-culturable as well as non-viable microbial particles and their fragments, measuring only a small part of available immunologically reactive propagules. The obtained quantitative and qualitative results are additionally biased by the sampling time and numerous environmental, spatial or temporal alterations. In this study, the microbial aerosol sampling was limited in time (5 min) and space (stationary sampling only). Therefore, all these limitations should be kept in mind as they may, to some extent, underestimate the real environmental exposure.

## Conclusions

The results of this research conducted in Szczawnica sanatorium and in Bochnia Salt Mine health resort clearly indicate that the microbiological quality of the air is a key factor for their therapeutic and prophylactic functions. Especially for the patients with respiratory diseases, inhalation therapy with both naturally and artificially created aerosols can be very beneficial in the healing process, helping to mobilize their immune system to fight the disease. In this type of ‘special purpose’ premises, the measures that enable reducing microbial contamination should be introduced, especially if large groups of patients undergo such therapy. The promising simply solutions of this type may include among others: limitation on the number of patients who undergo therapeutic treatment at the same time, the regular hygienic control of therapeutic rooms regarding microbiological quality of the air and, adjusted to its results, the number of medical services provided to the patients as well as introduction of air-conditioning system to ensure a ‘clean’ air and to protect the healing properties of this type of environment.
